# Experimental Investigation of the Effect of Hydrogen on Fracture Toughness of 2.25Cr-1Mo-0.25V Steel and Welds after Annealing

**DOI:** 10.3390/ma11040499

**Published:** 2018-03-27

**Authors:** Yan Song, Mengyu Chai, Weijie Wu, Yilun Liu, Mu Qin, Guangxu Cheng

**Affiliations:** 1 School of Chemical Engineering and Technology, Xi’an Jiaotong University, Xi’an 710049, China; chaimengyu929@stu.xjtu.edu.cn (M.C.); wjwu629@stu.xjtu.edu.cn (W.W.); qinmu.1990.0113@stu.xjtu.edu.cn (M.Q.); 2 State Key Laboratory for Strength and Vibration of Mechanical Structures, Xi’an Jiaotong University, Xi’an 710049, China; yilunliu@mail.xjtu.edu.cn; 3 School of Aerospace, Xi’an Jiaotong University, Xi’an 710049, China

**Keywords:** welding, fracture toughness, hydrogen embrittlement, 2.25Cr-1Mo-0.25V

## Abstract

Hydrogen embrittlement (HE) is a critical issue that hinders the reliability of hydrogenation reactors. Hence, it is of great significance to investigate the effect of hydrogen on fracture toughness of 2.25Cr-1Mo-0.25V steel and weld. In this work, the fracture behavior of 2.25Cr-1Mo-0.25V steel and welds was studied by three-point bending tests under hydrogen-free and hydrogen-charged conditions. The immersion charging method was employed to pre-charge hydrogen inside specimen and the fracture toughness of these joints was evaluated quantitatively. The microstructure and grain size of the specimens were observed by scanning electron microscopy (SEM) and by metallurgical microscopy to investigate the HE mechanisms. It was found that fracture toughness for both the base metal (BM) and the weld zone (WZ) significantly decreased under hydrogen-charged conditions due to the coexistence of the hydrogen-enhanced decohesion (HEDE) and hydrogen-enhanced localized plasticity (HELP) mechanisms. Moreover, the formation and growth of primary voids were observed in the BM, leading to a superior fracture toughness. In addition, the BM compared to the WZ shows superior resistance to HE because the finer grain size in the BM leads to a larger grain boundary area, thus distributing more of the diffusive hydrogen trapped in the grain boundary and reducing the hydrogen content.

## 1. Introduction

Hydrogenation reactors are widely used in the petroleum refining industry. Due to the explosive nature of hydrogen, the reliability of equipment is an important factor that is taken into consideration for practical applications. In recent years, with increasing demand for higher operating temperatures and pressures and for greater resistance to the creep phenomenon, traditional Cr–Mo steel can no longer meet these stringent safety requirements. Therefore, there is a need for new materials that are suited for these applications. A new low-alloy steel 2.25Cr-1Mo-0.25V has superior mechanical properties, possesses high-strength, and is being employed in the manufacture of hydrogenation reactors due to its improved resistance to oxidation and hydrogen embrittlement (HE) [1,2,3].

The introduction of hydrogen in the manufacturing and operating processes causes a degradation in the mechanical properties of alloy steel and can easily lead to fracture failure [4,5]. Since hydrogenation reactors are exposed to an aggressive hydrogen environment, HE is a critical issue that diminishes the reliability of these methods [6]. Hence, it is of great significance to study the effect of hydrogen on the mechanical properties of alloy steel, both for a reliability assessment and the optimal design of components. There are many studies that have been performed on this topic. Briottet et al. studied the fatigue crack initiation and growth in Cr–Mo steel under hydrogen pressure and indicated that, with increasing hydrogen pressure, the number of cycles needed to initiate crack formation decreased [7]. Garcia et al. studied the effect of hydrogen on the tensile properties of CrMoV steels by means of the small punch test, and found that the CrMoV steel has a high susceptibility to HE [8]. Colombo et al. studied the fatigue behavior of hydrogen pre-charged low-alloy Cr–Mo steel, and indicated that the fatigue-crack growth rate of hydrogen pre-charged specimens is about two or three times higher while the fracture toughness is lower. In addition, it was also indicated that the electrochemical pre-charging method seems to be sufficiently conservative with respect to gaseous hydrogen exposure [9]. Pillot et al. studied the effect of hydrogen on mechanical properties of 2.25Cr–1Mo steel grades and showed that, with increasing hydrogen content, the charpy fracture toughness of 2.25Cr–1Mo decreased [10,11].

Welding is commonly employed in the fabrication of hydrogenation reactors. The welded joint is considered as one of most critical parts in these reactors. Thus, in practice, after welding processes, a heat-treatment, i.e., annealing, is applied to improve the mechanical properties of the welded joint. However, since the hydrogenation reactor is operating under extremely high pressures and temperatures, cracks generated in the welded joint part (e.g., at the base metal (BM) and in the weld zone (WZ)) can compromise the performance of the reactor. Therefore, the fracture failure of the welded joint part is still a major concern for hydrogenation reactors [12,13]. The investigation on fracture toughness of these welded joints has a significant contribution in evaluating the safety of hydrogenation reactors [14,15]. Tanaka et al. studied the fracture toughness of CrMoV under high temperature by small punch testing [16]. Jiang et al. studied the evolution of microstructure and mechanical properties of 2.25Cr-1Mo-0.25V steel with different initial microstructures during tempering [2]. Guo et al. studied the correlation between microstructure and fracture toughness in an advanced 9Cr/CrMoV dissimilarly welded joint [17].

However, to the best of our knowledge, the effect of hydrogen on fracture toughness of 2.25Cr-1Mo-0.25V steel and welds has not been studied before. In this work, the fracture toughness of a 2.25Cr-1Mo-0.25V steel-welded joint (i.e., at the BM and in the WZ) was tested by three-point bending experiments. To investigate the effect of hydrogen on fracture toughness, half of the specimens were pre-charged with hydrogen via an immersion charging method. Hydrogen concentrations in BM and WZ specimens were measured by LECO-TC-600. To study the HE mechanisms, the microstructures and fracture morphology of specimens were investigated by metallurgical microscopy and scanning electron microscopy (SEM), respectively.

## 2. Experimental Details

### 2.1. Materials and Specimens

**Figure 1:**
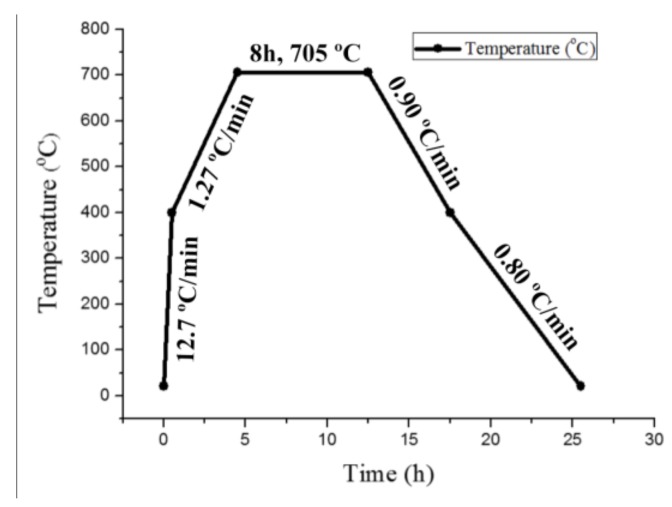
Post-weld heat treatment procedure.

ArcelorMittal Company (Luxemburg, Luxembourg) provided the 2.25Cr-1Mo-0.25V steel. The 2.25Cr-1Mo-0.25V steel base metal was treated by normalizing and tempering thermal treatments, with the temperatures for these two heat treatments are 910 °C and 720 °C, respectively. Steel plates with a thickness of 98 mm, a width of 320 mm, and a length of 800 mm were joined by the narrow-gap welding process by Lanzhou LS Heavy Equipment Co., Ltd. (Lanzhou, China). During the welding process, CM-A106HD wire was used for Shielded Metal Arc Welding for root welding, the Submerged Automatic Arc Welding was performed for the remaining passes with US-521H as a filler metal and PF500 as a flux. The welding current, arc voltage, and travel speed were maintained at 500 A, 32 V, and 22 mm/min, respectively. The type of weld is V. Following this step, post-weld heat treatment (PWHT, i.e., annealing) was performed to eliminate any residual stress and improve the stability of the microstructure. The welded pads were treated at a temperature of 705 °C for 8 h and then cooled to room temperature at a rate of 0.9 °C min^−1^, as shown in Figure 1. The welded joints were also analyzed using X-radiography for locating any defects. The chemical composition of BM and WZ are shown in Table 1 and Table 2, respectively.

**Figure 2:**
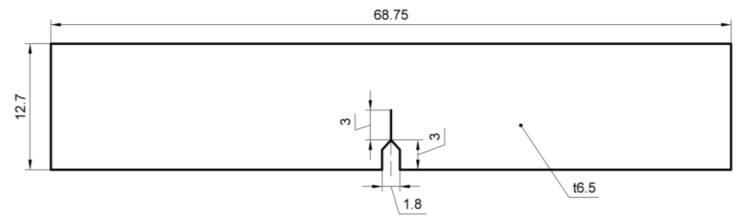
Dimension of the specimen. All units are in mm.

**Figure 3:**
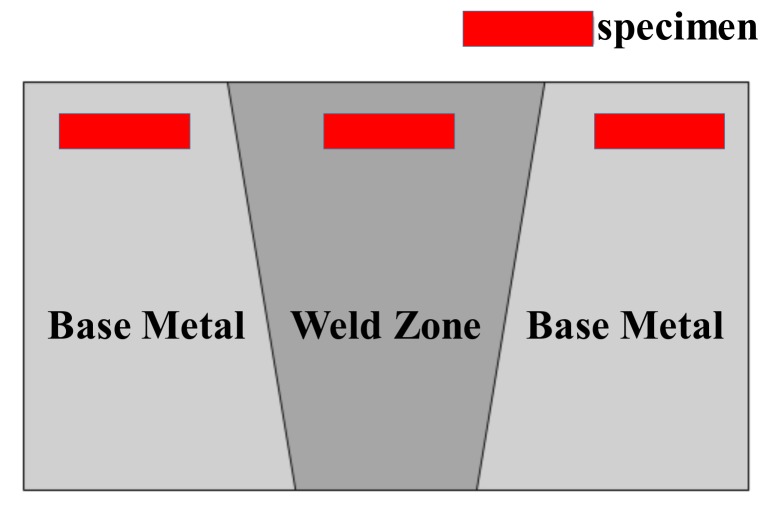
Schematic of positions of the specimens’ sampling.

Single edge notched bend (SENB) specimens with a thickness of 6.5 mm were machined from the top of welded joints to determine the fracture toughness in accordance with ISO 12135 standard [18] and the ASTM E1820 standard [19]. Notches were located in BM and WZ, as shown in Figure 2. The position of the specimens’ sampling zone were shown in Figure 3. The specimens were gradually ground with different grades of grinding papers up to 1500 grit and then mirror-polished using a 1 μm diamond paste. After the grinding step, the samples were cleaned with de-ionized water and acetone, and finally dried with the help of a stream of cold air. The microstructures of the BM and the WZ were then characterized by metallurgical microscopy. A 5% nital solution was used as an etchant, and specimens were immediately cleaned with methanol. 

### 2.2. Hydrogen Charging Process

Hydrogen charging was performed using the NH4SCN immersion charging method at room temperature. This method has been previously used for the HE tests of various materials including the Cr–Mo steel [20]. Additionally, the immersion charging method possesses higher efficiency and more simplicity in comparison with the other hydrogen charging methods [21]. In this work, the specimens were first fatigue pre-cracked to achieve a/W ≈ 0.5 (a is the total length of the notch starter plus the fatigue crack, and W is the width of the specimen), where the maximum peak force was 4 kN, and the stress ratio (minimum peak force/maximum peak force) was set to 0.1. Subsequently, the pre-cracked BM and WZ specimens were both immersed into a 20 wt % ammonium thiocyanate (NH4SCN) solution with a pH of 4.8 for 96 h. The specimens were then removed from the solutions and immediately tested. The escape of hydrogen during the tests was neglected as the time between the removal of the specimens from solutions and the end of fracture toughness tests was less than 20 min. Hydrogen concentration in the BM and WZ specimens were measured by LECO-TC-600 (Saint Joseph, MI, USA).

### 2.3. Fracture Toughness Test

Fracture toughness tests were carried out on a material testing machine (model: MTS 880/25t, Eden Prairie, MN, USA) according to ISO 12135 standard and the ASTM E1820 standard. The loading speed was set to 0.5 mm/min and the load–displacement curve was recorded for calculation of the *J* integral. The tested specimens were subjected to high frequency fatigue to facilitate crack formation for the convenience of fractography investigation. There are 4 specimens for each condition (i.e., the BM, the WZ, and hydrogen-charged BM and WZ) that have been carried out. Fracture surfaces of the tested specimens were investigated using a scanning electron microscope (model: SU 3500, Krefeld, Germany).

## 3. Results

### 3.1. Microstructure and Grain Size

**Figure 4:**
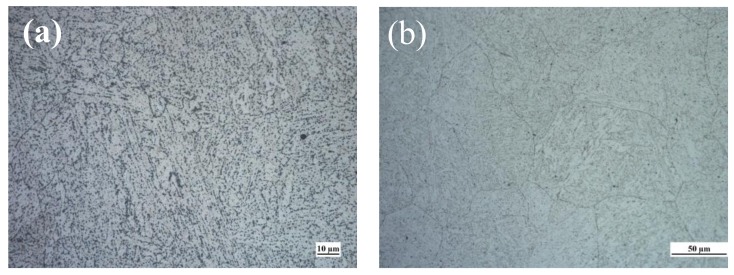
Microstructures of 2.25Cr1.Mo0.25V steel welded joint for (**a**) the BM and (**b**) the WZ.

**Figure 5:**
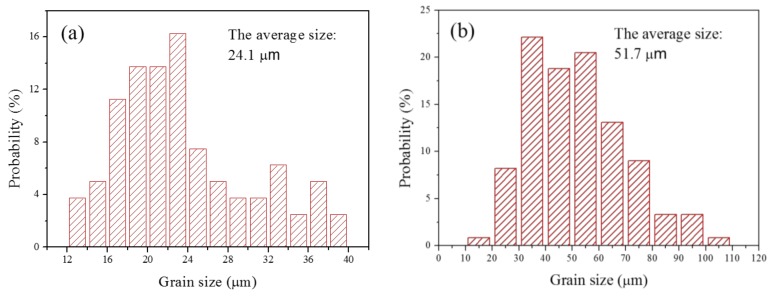
Grain size distribution of (**a**) the BM and (**b**) the WZ.

Figure 4 shows the typical microstructures of the BM and WZ. It is evident that both microstructures are composed of bainitic microstructures, and the bainite grain in the case of the WZ is larger than that of the BM. The grain sizes were determined by Image-Pro Plus software using at least 20 metallographic figures. The grain size was defined as the equivalent circle diameter of the grain. Figure 5 shows the probability distribution of grain size. The average grain sizes of the BM and the WZ were obtained as 24.1 and 51.7 μm, respectively (see Figure 5).

### 3.2. Fracture Toughness

**Figure 6:**
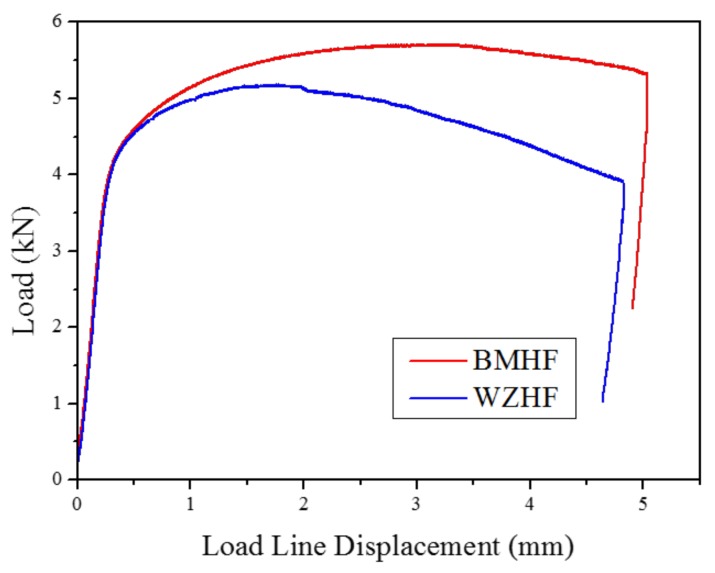
Typical load vs. load line displacement curves of hydrogen-free (HF) specimens.

Figure 6 shows the load–displacement curves of hydrogen-free specimens for BM and WZ produced from the fracture toughness tests. In both cases, a distinct two-stage feature was observed involving the elastic deformation stage and plastic deformation stage. This result indicates that the fracture mode in BM and WZ is ductile in nature. Even though the fracture mode is ductile in the two cases, significant differences exist in their properties. For the BM specimen, a pronounced load plateau is observed due to prominent plasticity-induced strain-hardening as well as blunting of the newly created crack tip. As the crack propagates, there is a minor decrease in the load, which finally drops down due to crack destabilization. However, for the WZ specimen, no apparent load plateau is observed and only a minor displacement is recorded before the maximum load is reached. Further crack growth causes the load to decrease continuously after the maximum load is passed. The maximum load of the WZ is obtained to be lower than that of the BM specimen. Therefore, these results indicate that the fracture toughness of the BM is higher than that of the WZ.

**Figure 7:**
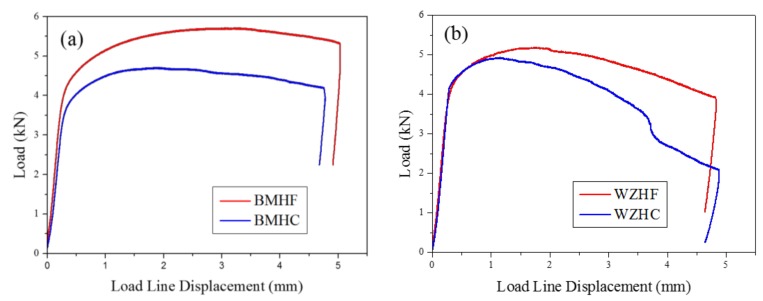
Typical load vs. load line displacement curves of HF and hydrogen-charged (HC) specimens for (**a**) the BM and (**b**) the WZ.

Similar displacements of load head were observed for hydrogen-free and hydrogen-charged conditions, indicating a similar stable crack propagation distance, as shown in Figure 7. However, for both BM and WZ specimens, the maximum loads under hydrogen-free conditions are higher than those under hydrogen-charged conditions. As a result, the fracture toughness of specimens under hydrogen-free conditions is superior to that under hydrogen-charged conditions. The effect of hydrogen on the fracture toughness of both the BM and the WZ can be elucidated by the fracture morphology and microstructure, which is discussed in the following section.

The elastic–plastic fracture mechanics approach is applied for the quantitative estimation of the fracture toughness. The *J* integral is obtained after splitting up its elastic component and plastic component and is expressed as

(1) $$J=J_{el}+J_{pl}$$

where the elastic component is related to the stress intensity factor *K* by

(2) $$J_{el}=\frac{K^{2}\left(1-\nu^{2}\right)}{E}$$

*K* can be estimated according to the following equation, in accordance with the ASTM E1820 standard,

(3) $$K=\left[\frac{PS}{BW^{3/2}}\right]f\left(a_{0}/W\right)$$

where *P* is the maximum load gained from the load–displacement curve, *S* is the specimen span, *B* is the thickness, *W* is the width of specimen, *a*_0_ is the initial crack length, ν is Poisson’s ratio, which equals 0.3, and *E* is Young’s modulus, which equals 210 GPa. $f\left(a_{0}/W\right)$ is the coefficient of stress intensity factor that is calculated by the following expression:

(4) $$f\left(a_{0}/W\right)=\frac{3{\left(a_{0}/W\right)}^{1/2}\left[1.99-\left(a_{0}/W\right)\left(1-a_{0}/W\right)\times \left(2.15-3.93\left(a_{0}/W\right)+2.7{\left(a_{0}/W\right)}^{2}\right)\right]}{2\left(1+2a_{0}/W\right){\left(1-a_{0}/W\right)}^{3/2}}$$

**Figure 8:**
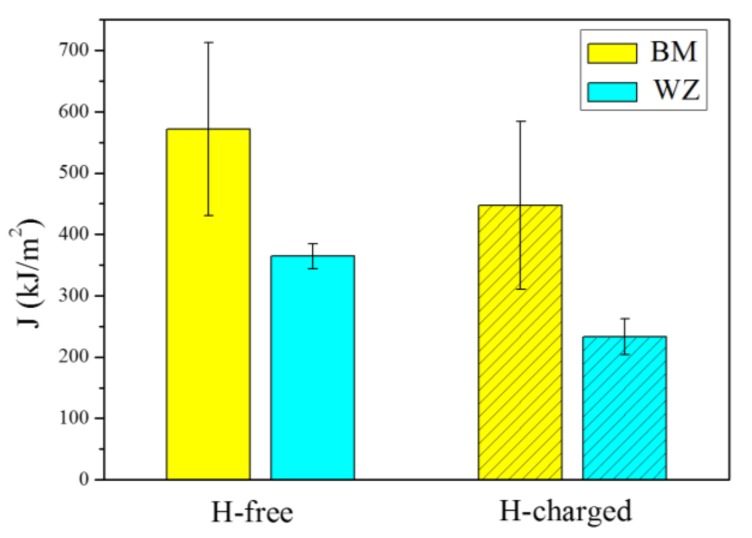
Fracture toughness *J* integral of hydrogen-free and hydrogen-charged specimens.

The plastic component of *J* is calculated by the following equation:

(5) $$J_{pl}=\frac{2A_{pl}}{B\left(W-a_{0}\right)}$$

where *A_pl_* is the plastic component of the deformation energy and is defined as the area under the load–displacement curve excluding the elastic part. The *J* integral obtained from the above equations is the fracture resistance at the first attainment of a maximum load plateau for fully plastic behavior. From the *J* integral evaluation, it is evident that the fracture toughness of BM is higher than that of the WZ, as shown in Figure 8. The average *J* integrals of the BM and the WZ under hydrogen-free conditions were determined to be 571.4 and 364.4 kJ/m^2^, respectively. Previous investigations have shown that the critical fracture toughness *J_IC_* estimated following the ASTM E1820 standard in 2.25Cr-1Mo-0.25V steel is 555 kJ/m^2^ [22,23], which is consistent with the present results. On the other hand, the presence of dissolved hydrogen leads to a significant reduction of fracture toughness. The average *J* integrals of the BM and WZ under hydrogen-charged conditions decreased to 447.5 and 232.8 kJ/m^2^, respectively. To quantify the HE resistivity, the parameter of sensitivity to hydrogen is defined as

(6) $$\delta_{H}=\frac{J-J_{H}}{J}\times 100\%$$

where *J* and *J_H_* are the average *J* integrals obtained after the fracture toughness of the hydrogen-free and hydrogen-charged specimens was tested, respectively. The calculated *δ_H_* values are 21.6% and 36.1% for the BM and WZ, respectively, indicating that the BM has a higher resistance to hydrogen than that of the WZ. Such a behavior is mainly caused by the microstructural change associated with the grain size, which is elaborated in the next section. It is emphasized that nondestructive evaluation on the WZ should be given high attention during the operation and maintenance of the hydrogenation reactor due to the lower fracture toughness and resistance to hydrogen of the WZ.

### 3.3. Fracture Morphology

**Figure 9:**
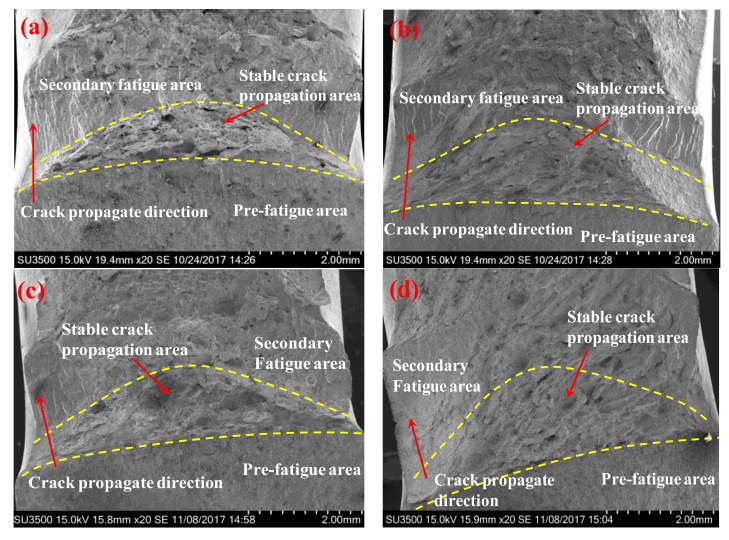
The macroscopic fracture surface including the pre-fatigue area, the stable crack propagation area, and the secondary fatigue area. Hydrogen-free conditions: (**a**) BM; (**b**) WZ. Hydrogen-charged conditions: (**c**) BM; (**d**) WZ. The dashed lines distinguish different regions on the fracture surface.

In order to understand the fracture mechanisms, the fracture morphology of specimens for the hydrogen-free and hydrogen-charged conditions was characterized by SEM. The macroscopic features of fracture surfaces are shown in Figure 9. It is evident that the fracture surfaces of all specimens are composed of three regions, i.e., the pre-fatigue area, the stable crack propagation area, and the secondary fatigue area. The stable crack propagation areas under the hydrogen-free conditions are rougher than those under the hydrogen-charged conditions. The interfaces between the stable crack propagation area and secondary fatigue area of the hydrogen-free specimens are more obvious than those of the hydrogen-charged specimens. These results suggest the presence of more brittle features in the hydrogen-charged specimens.

**Figure 10:**
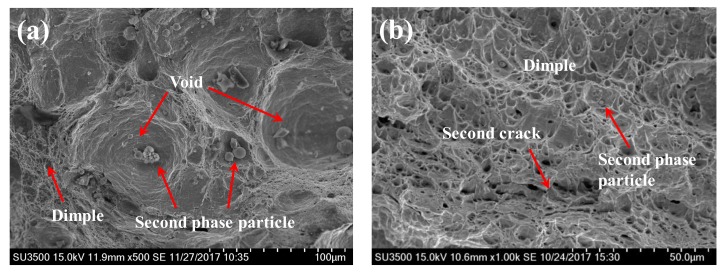
The fracture morphology of BM (**a**) and WZ (**b**) under hydrogen-free conditions showing ductile fracture.

Figure 10 displays the fracture morphology of BM and WZ specimens under hydrogen-free conditions. It is obvious that the fracture processes are characteristics of the ductile fracture with microvoid nucleation, growth, and coalescence. The fracture surface of BM consists of several large primary voids (30–60 μm) and numerous dimples with small size. Due to high local stress, there is a tendency for the growth of the phase boundary, the grain boundary, and the second-phase particles and for the aggregation of adjacent cracks to generate voids and dimples [24,25]. As a result, several second-phase particles and inclusions are found in the center of primary voids and dimples. The fracture surface of the WZ shows a similar ductile fracture morphology consisting of a large number of dimples, which generally originate from the second-phase particles. However, no primary voids with large size are found in the WZ case. Only massive dimples with small sizes (1–4 μm) formed during the fracture process. Cao et al. [17] demonstrated that, in the fracture of a high-strength steel, more plastic strain and energy is consumed leading to dimples with larger sizes. As a result, the formation and growth of large-sized primary voids in the BM, compared to those in the WZ, leads to a higher resistance to crack propagation. The appearance of secondary cracks also causes a reduction in the resistance to crack propagation of the WZ, as shown in Figure 10b. Therefore, the large-sized primary voids and secondary cracks contribute to the different fracture toughness observed in the BM and the WZ.

**Figure 11:**
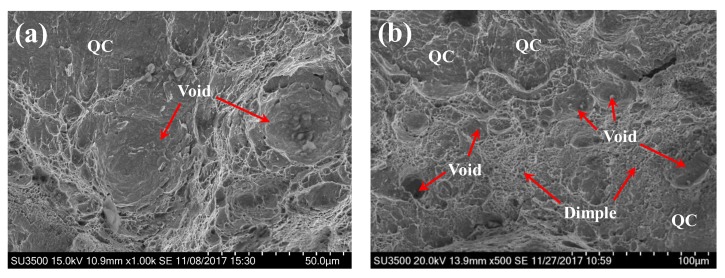

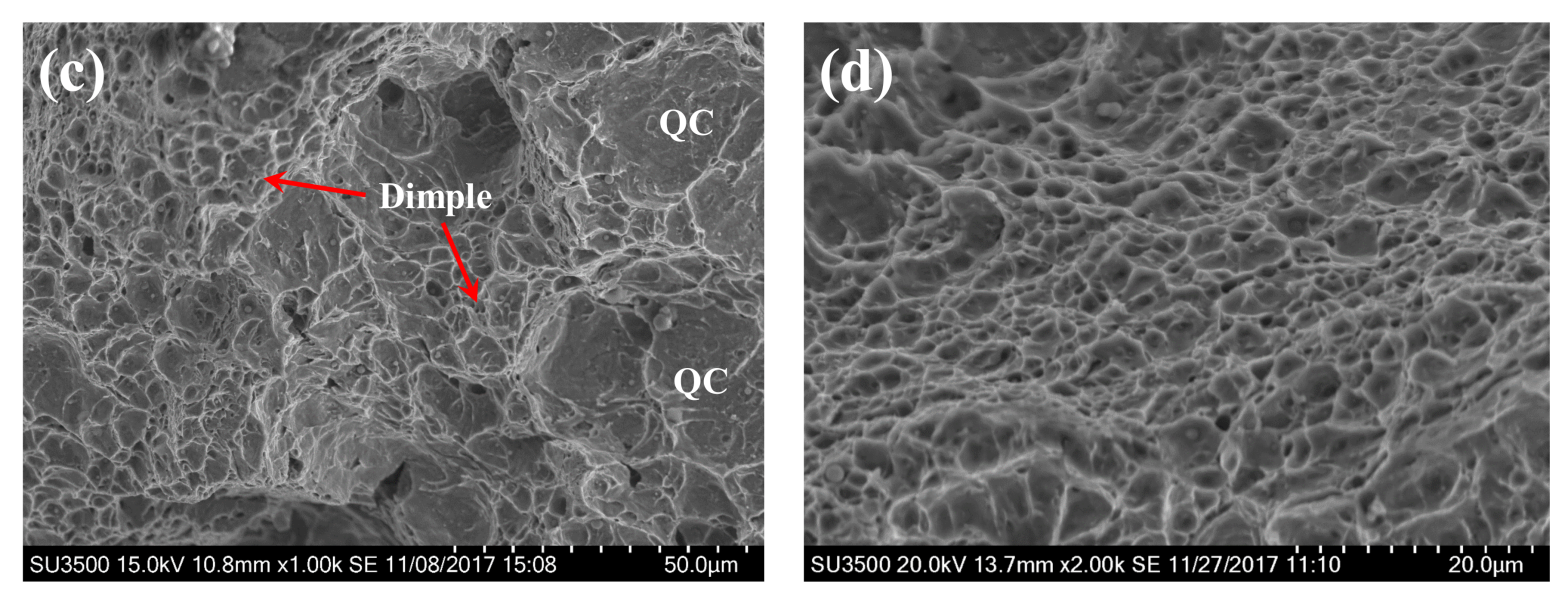
The fracture morphology of the BM (**a**,**b**) and the WZ (**c**,**d**) under hydrogen-charged conditions showing brittle and ductile fractures (**a**–**c**) and fine dimples (**d**).

In contrast to the hydrogen-free case, the fracture morphology of the BM and WZ specimens under hydrogen-charged conditions show mixed ductile and brittle features, as shown in Figure 11. A large number of shallow dimples, voids, and quasi-cleavage (QC) facets are found on the fracture surface of the BM. The voids are much smaller (10–30 μm) in comparison to those under the hydrogen-free conditions, indicating a lower resistance to crack propagation. Quasi-cleavage (QC) is a brittle fracture mode similar to cleavage. A remarkable feature of QC is the presence of planar facets, but these facets do not occur along a known cleavage plane. The appearance of QC facets on the fracture surfaces of both BM and WZ specimens indicates a major brittle feature, suggesting local cleavage by the presence of hydrogen, as shown in Figure 11a–c. Figure 11d shows the ductile dimples on the fracture surface of WZ, where the smaller size of dimples is evident when compared to the hydrogen-free case. It has been reported that the mean diameter of dimples is a measure of toughness due to the fact that more dissipated energy is needed to generate larger coarse dimples [26]. The mean diameters of dimples for the WZ specimens under hydrogen-free and hydrogen-charged conditions were estimated to be 2.64 μm and 1.35 μm, respectively. The reduced size of dimples indicates a decrease in resistance to crack propagation. Therefore, the appearance of QC facets and the decreased size of voids and dimples resulted in a remarkable reduction in fracture toughness under hydrogen-charged conditions, as seen in Figure 8. Table 3 summarizes the current results involving the fracture toughness values, microstructural features, and fracture mechanisms.

## 4. Discussion

### 4.1. Mechanisms of Hydrogen Effects

HE is a phenomenon that causes loss in ductility or strength in the presence of sufficient hydrogen. Several articles have reported that the dissolved hydrogen could lead to significant diminishments of mechanical properties such as fracture toughness [26], strength [27], ductility [28], and fatigue crack growth resistance [9]. In the present investigation, the pre-charged hydrogen was found to cause a 21.6% and 36.1% decrease in fracture toughness for the BM and the WZ, respectively. Although there is consensus on the detrimental effect of hydrogen on mechanical properties of materials, there is still strong disagreement about the inherent nature of the HE mechanisms due to microstructural and external factors [29]. It has been determined that no single mechanism can completely explain all of the observed hydrogen-degraded effects [30]. Thus, the mechanisms of hydrogen effects in this study require in-depth explorations.

There are two well-established micro-mechanisms for HE in metallic materials. One is the hydrogen-enhanced decohesion model (HEDE) and the other is the hydrogen-enhanced localized plasticity model (HELP). The HEDE mechanism hypothesizes that hydrogen atoms reduce the cohesive bond energy between metal atoms, leading to a significant reduction of fracture energy and hence causing brittle crack propagation [31]. Although direct experimental measurements of cohesive bond energy are not available, there are indirect evidences supporting the HEDE effect, such as the appearance of intergranular fracture, cleavage-like fracture, and an initiation of microcracks [32]. On the other hand, the HELP mechanism is based on the observation that hydrogen atoms enhance the mobility of dislocations through an elastic shielding effect, and consequently increase the plastic deformation ahead of the advancing crack tip [33]. The HELP mechanism consists of a highly localized plastic fracture process caused by micro-void coalescence rather than a brittle failure. In short, HEDE is responsible for the brittle fracture, while HELP contributes to the ductile fracture. 

It is apparent that the concurrent contribution of HEDE and HELP mechanisms is responsible for the remarkable reduction in fracture toughness in the BM and WZ specimens due to the simultaneous presence of brittle quasi-cleavage regions and locally ductile features (Figure 11). In this study, the hydrogen concentrations of BM and WZ specimens were measured to be 43 and 41.6 wppm, respectively. This indicated that the 96 h pre-charging process ensured enough dissolved hydrogen throughout the specimen, thereby leading to a high probability of activating both HEDE and HELP mechanisms. In the vicinity of the pre-fatigue crack tip, dissolved hydrogen mainly concentrates at trap sites due to lattice dilation by hydrostatic stress [26]. A critical concentration of trapped hydrogen at grain boundaries decreases the cohesive strength of the grain boundary and reduces the fracture initiation stress. During fracture toughness tests, brittle cracking is usually promoted when the crack tip opening stress exceeds the cohesive strength of the grain boundary. As a result, quasi-cleavage cracking regions are obtained on the fracture surfaces. As mentioned earlier, the appearance of brittle fracture could provide evidences for the HEDE effect despite the fact that it is very hard to measure the decrease in cohesive bond energy [31]. Thus, the HEDE mechanism is responsible for the occurrence of quasi-cleavage regions on fracture surfaces. On the other hand, the presence of voids with smaller size and shallow dimples in Figure 10a,b, and the fine dimples with smaller size in Figure 10d indicate that hydrogen-enhanced dislocation processes were active ahead of the crack tip for both BM and WZ specimens. In this case, the absorbed hydrogen ahead of the crack tip exceeds the critical hydrogen concentration, leading to a decrease in the critical stress and strain of void nucleation. The decrease in the formation energy of vacancy and free surfaces can cause an acceleration of void nucleation [34]. Therefore, the HELP mechanism contributes to the formation of voids and dimples with smaller sizes.

### 4.2. Resistance to HE

Although pre-charged hydrogen conditions resulted in a significant reduction in fracture toughness for both BM and WZ specimens, the details of their resistances to HE were different. For instance, BM specimens, compared with WZ specimens, showed a higher resistance to HE because a lower *δ_H_* is obtained. This is in spite of the facts that both BM and WZ specimens had similar hydrogen concentrations and that the hydrogen-charging processes were performed in the same solution environment for an identical time duration. This is mainly attributed to the fine-grained microstructure of BM. It has been proposed that microstructural features such as grain boundaries and dislocations can not only trap hydrogen but also serve as pathways for the diffusion of hydrogen [35]. In this study, the microstructure analysis reveals that the average grain size of the BM is 24.1 μm, while the WZ shows an average grain size of 51.7 μm, as shown in Figure 5. The finer grain size of BM results in a larger grain boundary area, consequently distributing more of the diffusive hydrogen trapped in the grain boundary, thereby reducing the hydrogen content per unit grain boundary area. Therefore, the BM specimen with finer grain size exhibits a lower sensitivity to HE than the coarse-grained WZ. Previous investigations have also shown an improvement in resistance to HE by decreasing the grain size in high-strength low-alloy steels [36] and Fe–Ni-based alloys [37], which is consistent with the current results.

However, some disparities in the literature still exist. For example, Mine et al. [38] showed that decreasing the grain size from 265 to 85 nm in austenitic stainless steels did not lead to an increase in resistance to HE. Malitckii et al. [39] reported complex and non-linear HE sensitivity behavior in ferritic stainless steel. It was concluded that the change in grain size had a minor effect on HE sensitivity, whereas the microstructural features associated with ferrite decomposition and secondary-phase formation primarily led to an improvement of HE sensitivity. Therefore, further studies need to be performed to comprehensively investigate the exact relationship between the microstructure and resistance to HE by considering different microstructural variations and by improving the accuracy of hydrogen concentration measurements.

## 5. Conclusions

In this study, the effect of hydrogen on fracture toughness behavior of 2.25Cr-1Mo-0.25V steel and welds was investigated. Three-point bending tests were carried out on BM and WZ specimens under hydrogen-free and hydrogen-charged conditions, and the *J* integral was evaluated for quantitatively assessing fracture toughness. Additionally, the mechanisms of the effect of hydrogen and the resistance to hydrogen were studied by microstructural analyses. Major conclusions are stated as follows:

The BM specimen showed superior fracture toughness than the WZ in 2.25Cr-1Mo-0.25V welded joints due to the formation and growth of primary voids in the BM.

The presence of dissolved hydrogen resulted in a significant reduction in fracture toughness for both BM and WZ specimens. The coexistence of the HEDE and HELP mechanisms is proposed to be responsible for this reduction due to the simultaneous presence of brittle quasi-cleavage regions and localized ductile features on the fracture surfaces of hydrogen-charged specimens.

The BM specimen, compared with the WZ specimen, exhibited a superior resistance to HE. This was attributed to the fact that a finer grain size in the BM resulted in a larger grain boundary area, thus distributing more of the diffusive hydrogen trapped in the grain boundary and reducing the hydrogen content per unit of grain boundary area. Therefore, it is proposed that grain size refinement effectively enhances resistance to HE.

## Figures and Tables

**Table 1:** Chemical composition of the 2.25Cr-1Mo-0.25V base metal (BM) (wt %).

Element	C	Si	Mn	P	S	Cr	Mo	V	Al
Percentage	0.15	0.1	0.54	0.009	0.01	2.3	0.98	0.3	0.05

**Table 2:** Chemical composition of the 2.25Cr-1Mo-0.25V weld zone (WZ) (wt %).

Element	C	S	P	Si	Mn	Cr	Ni	Mo	Cu	V
Percentage	0.12	0.004	0.004	0.22	1.07	2.45	0.03	1.03	0.11	0.42

**Table 3:** Summary of current results.

Specimen	Average Grain Size (μm)	Hydrogen Condition	Average Fracture Toughness (kJ/m^2^)	SEM Features	Fracture Mechanisms
BM	24.1	H-free	571.4	voids and dimples	ductile fracture
BM	24.1	H-charged	447.5	voids, dimples and quasi-cleavage facets	ductile and brittle fracture
WZ	51.7	H-free	364.3	dimples	ductile fracture
WZ	51.7	H-charged	232.8	dimples and quasi-cleavage facets	ductile and brittle fracture
